# Latino and Non-Latino Perceptions of the Air Quality in California’s San Joaquin Valley

**DOI:** 10.3390/ijerph13121242

**Published:** 2016-12-14

**Authors:** Paul Brown, Linda Cameron, Ricardo Cisneros, Rachel Cox, Erin Gaab, Mariaelena Gonzalez, Steven Ramondt, Anna Song

**Affiliations:** Health Sciences Research Institute, University of California, Merced, CA 95343, USA; lcameron@ucmerced.edu (L.C.); rcisneros@ucmerced.edu (R.C.); jmarks4@ucmerced.edu (R.C.); egaab@ucmerced.edu (E.G.); mgonzalez82@ucmerced.edu (M.G.); sramondt@ucmerced.edu (S.R.); asong5@ucmerced.edu (A.S.)

**Keywords:** air quality, perceptions, Latino health

## Abstract

The San Joaquin Valley (SJV) of California has poor air quality, high rates of asthma, and high rates of obesity. Informational campaigns aimed at increasing awareness of the health impacts of poor air quality and promoting behavior change need to be tailored to the specific target audiences. The study examined perceptions of air quality, perceived health impacts, and methods of accessing information about air quality between Latinos and other groups in the SJV. Residents of the SJV (n = 744) where surveyed via one of three methods: community organizations (256), public locations (251), and an internet panel (237). The results suggest that people perceive the air quality in their region to be generally unhealthy, particularly for sensitive groups. The air quality is more likely to be reported as being unhealthy by people with health problems and less unhealthy by Latinos and people who report regularly exercising. Latinos are more likely to report working outdoors regularly, but also more likely to report being able to reduce their exposure if the air quality is unhealthy. The results report differences in informational sources about air quality, suggesting that informational campaigns should target high risk groups using a variety of media.

## 1. Introduction

The cities in the San Joaquin Valley (SJV) of California are consistently rated as among those with the worst air quality [1] in the nation. In 2015, there were over 38 days when air quality in Fresno was deemed “unhealthy” and individuals were advised to avoid outdoor activities. The air pollution has been linked to high rates of asthma and other respiratory diseases [2], particularly among the region’s most vulnerable populations [3]. The region is distinctive in its high levels of income disparities as well as its ethnically diverse communities, with 49% of the population reporting as Latino, 38% White, and 8% Asian, and poverty levels that are comparable to the poorest regions of Appalachia [4,5]. In addition to health issues related to air quality, the SJV also has some of the highest rates of obesity [6] and diabetes [7] in California. This creates a challenge for public health officials in the region, namely how to help people get accurate information on the air quality and to identify times when it was healthy to be outdoors and exercise, thereby promoting physical activity and engagement in outdoor activities.

Promoting healthy exercise and engagement in outdoor activities during times when air pollution levels are low requires a series of conditions: (1) the public needs to be aware that engaging in physical activity when air quality is poor is unhealthy; (2) people should understand how to identify whether the air quality is healthy at a particular time of day; and (3) the public must have the capacity to adjust their schedules so they can be active outdoors when the quality is good and stay indoors when the quality is poor. There are a number of air quality indicators to help people monitor the air quality in the SJV. These include traditional media sources (e.g., regional newspapers, television, and radio), a Real-time Air Advisory Network (RAAN) that provides information from monitoring stations around the region [8], and an air quality flag program that enlists schools and other organizations to post a flag showing the air quality for the day [9,10]. However, studies in other areas of the world, including Texas, Oregon, New Jersey, Pennsylvania, Northeast England, Kenya, and Korea [11,12,13,14,15,16,17,18], have found that individuals’ reports on perceptions of air quality are often inaccurate and the perceived link between air quality and health is often not well understood. It is unclear whether the at-risk residents of the SJV are monitoring air quality, understanding the link between air quality and health, and adjusting their levels of outdoor activities accordingly.

Informational campaigns aimed at increasing awareness of the health impacts of poor air quality and promoting behavior change need to be tailored to the specific target audiences [19]. The purpose of this study was to survey residents in the SJV regarding their perceptions of air quality, the sources of their information, and whether they report altering their behavior based on the air quality information. The survey was developed and conducted in partnership with a Community Advisory Group whose input included identifying air quality as a primary concern requiring translational research; vetting the survey questions; advising on the methods of survey administration so as to target hard to reach, vulnerable populations in the region; and interpreting the results. The aim is to identify systematic differences in perceptions of air quality, perceived health impacts, and methods of accessing information, particularly differences between Latinos, people of other ethnicities, and Whites in the region. Because previous studies have found the impacts of poor air quality are especially pronounced on Latino communities [20,21,22], the study tests whether perceptions and impact of air quality vary across ethnic and demographic groups in the SJV and the extent to which those most impacted by air quality are able to curtail or avoid exposure when the air is unhealthy.

## 2. Methods

### 2.1. Sample: Three Approaches Were Used to Survey Residents of the SJV

Community meetings. Based on the recommendations of the Community Advisory Group, members of the research team visited community organizations (e.g., Boys Club, the Latino Community Roundtable, etc.) during regularly scheduled meetings and administered the survey to consenting participants. Participants could choose to complete either an English version or a Spanish version of the questionnaire.

Public locations. The Community Advisory Group identified locations in the region (e.g., outdoor markets, local malls, public parks) that vulnerable populations were likely to access. Teams of research assistants recruited and administered the survey to consenting participants. Participants could choose to complete either an English version or a Spanish version of the questionnaire.

On-line survey. In order to access participants from across the broader SJV, a survey was distributed to members of an on-line survey panel recruited by a survey company. The survey was restricted to residents of the SJV (via the zip code of their home address) and targeted both English and Spanish speaking participants. Participants could choose to complete either an English version or a Spanish version of the questionnaire. 

Ethics approval was obtained from the University of California, Merced Institutional Review Board (UCM14-0033).

### 2.2. Survey Items

The survey included the following items relating to the respondents’ views of air quality in their region and the SJV:

1. “In the past month, what was the air quality like in (your area)?” (very unhealthy, unhealthy, unhealthy for sensitive groups, moderately healthy, good);
2. “In the past month, what was the air quality like in the other areas of the San Joaquin Valley?” (very unhealthy, unhealthy, unhealthy for sensitive groups, moderately healthy, good);
3. In a normal year, what is the air quality like in (your area) in the … Summer? Winter? Spring? Fall? (very unhealthy, unhealthy, unhealthy for sensitive groups, moderately healthy, good).

The survey asked about how often the exercised and worked outdoors, and whether they could avoid being outdoors when the air quality was poor.

4. “In past month, how often did you go outside to exercise on average?” (never, 1 or 2 times per week, 3 or 4 times per week, 5 or 6 times per week, or everyday);
5. “In the past month, how many days did you work outside for more than 30 min on average?” (never, 1 or 2 times per week, 3 or 4 times per week, 5 or 6 times per week, or everyday);
6. “In the next month, on days when the air quality is bad, how often will you avoid exercising for long periods of time?” (never, almost never, sometimes, often, every time);
7. “Do you have a job that requires you to work outside regardless of the air quality?” (yes or no);
8. “Do you have to run errands or do other daily activities that require you to be outside regardless of the air quality?” (yes or no).

The perceived impact of air quality on their own and their family member’s health was assessed by the following items:

9. “In the past month, did you have any health problems that were made worse by the air quality?” (yes or no);
10. “Have you ever been told by a doctor that you have asthma?” (yes or no);
11. “For family members who are living with you now: Have any of them been told by a doctor that they have asthma?” (yes or no);
12. “In the past month, did any family members who are living with you have any health problems that were made worse by the air quality?” (yes or no);
13. “What do you think the chances are of having problems with your health because of the air quality in this region?” (almost zero, very small, small, moderate, high, very high, almost certain);
14. If you continue to live in this region, what do you think the chances are that the air quality will lead to health problems at some time in your life? (almost zero, very small, small, moderate, high, very high, almost certain).

The respondents were asked to indicate (yes or no) whether how they determine the air quality in the region:

15. “What do you do to decide whether the air quality is good?” (Look outside or at the sky? Look to see how clearly you can see mountains? Check reports on TV? Check reports on the radio? Look online or on the internet? Use a phone app? Check the smell of the air? Look at the air quality flags in front of public buildings? Check the Air Quality Index in the newspaper?).

Finally, the survey asked for demographic information, including age, gender, education, household income, and ethnicity.

### 2.3. Analysis

Descriptive statistics (means, frequencies) were used to identify general patterns of responses regarding air quality perceptions, sources of air quality information, health problems attributed to poor air quality, and changes in outside physical activity when the air quality is poor. The impact of air quality on the respondent’s own health was determined by whether they responded “yes” to either having had their health impacted by air quality in the past month or that they had been diagnosed with asthma. The impact of air quality on a family member’s own health was determined by whether they responded “yes” to either having a family member whose health was impacted by air quality in the past month or had been diagnosed with asthma. 

Regression analyses were used to identify the predictors of perceived air quality, including demographic characteristics, health impact of air quality in their region (city) and in the SJV; the perceived impact of air quality on their health; the amount they currently exercise and work outdoors each week; and the extent to which they can avoid exercise, working outdoors, or running errands if the air quality was poor. Regression analysis was conducted to test whether there are differences between ethnicities; whether any differences can be explained by educational status, health issues (own or family), and whether they currently exercise each week. The results report the results from the regression analysis in two ways: With only age, gender and ethnicity, and then with age, gender, ethnicity and the other predictors. Finally, the results report the most commonly used methods for determining the air quality and whether there are differences between ethnicities in the sources of information. Because the survey was administered using three sampling strategies (i.e., through recruiting participants at community meetings, in public places, and via the internet), the analyses control for sampling strategy using mixed linear models that account for nested responses.

## 3. Results

### 3.1. Sample

Data were collected from 744 individuals, including 256 from community organizations, 251 from public locations, and 237 through the Internet panel. The characteristics of the sample are shown in Table 1.

The average age of the entire sample was 42 years, which is similar to the average age of the population of the SJV (40 years old). Participants accessed from community organizations tended to be older (average age of 49) and female (75% of respondents) compared with those recruited from public places (average age of 33, 57% female) and over the Internet (average age of 42, 63% female). The entire sample had more self-identified Whites and fewer self-identified Latinos (37% and 42%, respectively) compared with population in the region (32% and 49%, respectively), with the community organization and Internet respondents tending to only slightly underrepresent Latinos (38% and 49%, respectively). For the purposes of subsequent analysis, comparisons are made between self-identified Latinos and all other ethnicities compared with Whites. 

### 3.2. Perceptions of Air Quality

Overall, 28% of the respondents reported that the air quality in their town or the SJV is unhealthy or very unhealthy (Table 2). The respondents perceived the air quality to be worse in the summer months (35% reported air quality as unhealthy or very unhealthy) than in the spring (21%), winter (19%), or fall (24%) months. 

Regression analysis (Table 3) revealed that females perceived the air quality as being more harmful both in their area and for the SJV as a whole (columns 1 and 3 of Table 3). The effect was somewhat mitigated by the inclusion of education, whether they or a family member had a health condition related to air quality, and whether they exercised (columns 2 and 4). Latinos were generally more likely to report the air quality as being better in the region they lived in and in the SJV when compared with White respondents. This relationship was particularly evident when controlling for education, whether they or a family member had a health condition related to air quality, and whether they exercised regularly (columns 2 and 4). Taken together, these results suggest that people who exercise regularly tended to report better air quality in their region and in the SJV as a whole, those with health conditions or family members with health conditions tended to report worse air quality, Latinos tended to report better air quality, and females tended to report worse air quality in their region and the SJV. 

### 3.3. Perceptions of Impact of Air Quality on Own Health

As shown in Table 4 (column 1), there were no statistically significant differences in perceptions of air quality based on the age or ethnicity, although females tended to report higher levels of concern about the impact on their health. However, this relationship was mitigated by the inclusion of education, whether they or a family member had a health condition related to air quality, and whether they exercised (column 2). These results suggest that perceptions of the impact of air quality on health tend to increase with education and the presence of health conditions, but are lower for people who report regularly exercising.

### 3.4. Avoiding Exercise and Working Outdoors

As shown in columns 1 and 2 of Table 5, people who report regularly exercising or working outdoors were less likely to be older or female, while Latinos and people of other ethnicities were more likely to report working outdoors. Females, Latinos, and people of other ethnicities were more likely to report that they would avoid exercising if the air quality was poor (column 3). Latino respondents were less likely to report having a job that required that they work outdoors (column 4) and more likely to report being able to avoid running errands if the air quality was poor (column 4), but females reported being more likely to have an outdoors job. 

### 3.5. Sources of Information about Air Quality

The respondents’ reported sources of information are shown in Table 6. 80% reported that they get their information on air quality from looking outside, including whether they could see the mountains (73%). The most commonly reported medium for getting information was the TV. In contrast, the recommended ways of getting information—the Flag system or the Air Quality Index—were the least commonly cited sources (37% and 35%, respectively). There were differences in the sources of the information, with older people less likely to rely on whether they could see the mountains, check the TV, or check an air quality index, but more likely to look online, use a phone app, check the air quality flabs, or smell the air. Females were more likely to check the Air Quality Index, but less likely to check the Air Quality Flags. Latinos were more likely than Whites to check the Air Quality Flags, but less likely to check TV reports, check the radio, use a phone app, check the Air Quality Flags, or check the Air Quality Index.

## 4. Discussion

The study describes Latinos’ and non-Latinos’ perceptions of air quality in the SJV of California, the impact on their health, the extent to which they could take actions to avoid exposure when the air is unhealthy, and their current sources of information. The results suggest that, overall, less than a third of the people perceive the air quality in the region to be generally healthy, with some seasonality in their perceptions. Latinos were more likely to report higher perceived air quality than non-Latinos, but also as being more likely to be able to avoid exposure when the air quality is poor. Finally, the results suggest that the participants were more likely to rely on informal sources of information, such as seeing whether they could see the mountains, then the more precise and localized measures such as the Air Quality Index or the Air Quality Flags. 

The results from this study suggest that perceptions of the air quality were not related to education level, in contrast to the findings of some previous studies [18,23]. However, the participants of this study differed by ethnicity (Latinos reporting the air quality as generally less of a problem) and gender (females view air quality as more of an issue). This is generally consistent with previous findings [24] that reported people from vulnerable communities, such as Latinos, tend to suffer disproportionally the burden of the impacts of air pollution [25,26,27]. This suggests a need for more communication efforts targeting Latinos [20,21,22]. In contrast, those who reported that they exercised regularly also reported the air quality to be better. It is not known whether this is because they are less impacted by air pollution, lead healthier lifestyles, or whether they are minimizing the potential risk.

The results have implications for public health officials looking to increase the amount people exercise and go outdoors during times when the air quality is not harmful. 

The results suggest that, overall, only 1/3 of the public perceives the air quality as generally being unhealthy. They are correct in noting that the summer months tend to be the unhealthy (at least for ozone), but they understate the potential risk in the winter months when inversion layers can lead to bad air quality days (especially for particulate matter). One reason for the differences in seasons might the high reliance of people on sensory cues for attaining information about the air quality. Our results also suggest that the current methods for attaining information about the air quality is based on sensory cues rather than access official air quality monitoring information. This is likely to result in inaccurate assessments of the quality of the air, particularly for ozone and other pollutants that are not visible to the naked eye. Even though there is little flexibility in reducing exposure by delaying errands and other such tasks, only a 1/3 of the respondents’ report having a job that requires they work out outdoors. This suggests that there is the potential for campaigns that stress staying indoors when the air is unhealthy.

The current study can also inform effects in other regions across the United States. Among the American Lung Association’s list of the most polluted cities [1] in the United States, many listed regions are also ethnically diverse and/or economically challenged like California’s SJV (e.g., Las Cruces, NM; Johnstown, PA). Efforts in these other regions to reach marginalized populations who may not understand the link between air quality and health and may not have the ability avoid outdoor activities can be informed by these results.

The study has a number of limitations, including the reliance on self-reported exercise and activity data, the reliance on perceptions of air quality over the past month, the challenges with linking perceptions of air quality to actual exposure, and the reliance on a non-representative sampling frame. As a result, the results suggesting differences between groups in their perceptions of air quality and activity levels should be viewed as indications of the perceptions of the individuals, but not actually reflective of their behavior. These limitations are inherent in the retrospective nature of the survey and future studies should attempt to monitor perceptions and air quality on an ongoing basis. Still, a strength of the study is that it involved community engagement and a method of data collection that attempted to include individuals in the study who would normally be excluded. Previous studies have noted the challenges in recruiting members from vulnerable populations in regions such as the SJV [28,29,30]. The current study attempted to overcome this limitation by using three different methods—an internet survey (including Spanish speaking respondents), attendees at local community groups, and people in a number of public places around the region. This methodology has the potential to significantly improve access to and engagement with vulnerable communities in regions such as the SJV.

## 5. Conclusions

In conclusion, this study suggests that Latinos and non-Latinos in the SJV differ in their perceptions of air quality, their reported ability to avoid exposure, and the sources of information. Given the reliance on informal sources of information that are often inaccurate, the results suggest a need to identify new ways to deliver the information to the people in the region.

## Figures and Tables

**Table 1:** Demographics of study population.

	Study Population				Region (Census)		
Demographic Variable	Entire Sample	Internet	Public Places	Comm. Organizations	San Joaquin Valley	Merced County (Public Places)	Stanislaus County (Comm. Organizations)
Male	37.5%	45.1%	43.4%	24.6%	50.4%	50.3%	49.5%
Female	62.5%	54.9%	56.6%	75.4%	49.6%	49.7%	50.5%
Ethnicity:							
White	37.4%	48.5%	14.3%	49.6%	32.3%	26.5%	42.9%
Latino	42.3%	38.4%	57.8%	30.9%	48.6%	54.9%	41.9%
Black	4.6%	3.0%	6.4%	4.3%	5.0%	4.2%	2.9%
Asian	5.6%	5.9%	8.4%	2.7%	7.4%	8.1%	5.1%
Other	10.1%	4.2%	13.1%	12.5%	6.7%	6.3%	7.2%
Education:							
High School or below	51.9%	46.0%	55.8%	53.5%	51.5%	57.2%	48.5%
Some university, BA, or higher	48.1%	54.0%	44.2%	46.5%	48.5%	42.8%	51.5%
Age	42.1	42.4	32.9	49.1	40.1	38.4	41.9
n	744	237	251	256	3,971,659	255,793	531,997

**Table 2:** Perceived air quality in their region.

Perceived Air Quality in Their Region	Region					San Joaquin Valley
	In the Past Month	Summer	Fall	Winter	Spring	In the Past Month
Very unhealthy air quality	7.9%	10.2%	4.8%	4.7%	6.1%	6.0%
Unhealthy	20.3%	24.9%	16.2%	14.6%	17.8%	22.0%
Unhealthy for sensitive groups	30.2%	32.7%	36.8%	30.0%	33.1%	32.1%
Moderately healthy	33.6%	26.0%	33.6%	39.8%	33.0%	33.6%
Good air quality	8.0%	6.3%	8.5%	11.0%	10.0%	6.3%
n	699	704	703	707	703	685

**Table 3:** Predictors of perceived air quality in their region and in the San Joaquin Valley.

Variable/Statistic	Perceived Air Quality in Their Region		Perceived Air Quality in San Joaquin Valley	
	B (SE)	B (SE)	B (SE)	B (SE)
Intercept	3.47 * (0.16)	3.51 * (0.17)	4.44 * (0.15)	3.44 * (0.16)
Age	0.00 (0.00)	0.00 (0.00)	−0.01 ** (0.01)	0.00 (0.00)
Female	−0.23 * (0.08)	−0.15 ** (0.08)	−0.20 * (0.09)	−0.13 ** (0.08)
Latino	0.14 (0.09)	0.16 ** (0.10)	0.20 * (0.10)	0.26 * (0.09)
Other ethnicity	0.13 (0.10)	0.13 (0.12)	−0.02 (0.12)	−0.04 (0.11)
Education (High School or below)		−0.07 (0.08)		−0.13 ** (0.08)
Own health issue		−0.40 * (0.09)		−0.29 * (0.09)
Family members has a health issue		−0.32 * (0.09)		−0.21 * (0.09)
Exercise regularly		0.06 * (0.02)		0.07 * (0.02)
Public Places	−0.30 * (0.10)	−0.28 * (0.10)	−0.28 * (0.10)	−0.28 * (0.10)
Community Groups	−0.03 (0.10)	−0.07 (0.10)	0.03 (0.10)	−0.02 (0.10)
R^2^	0.03	0.12	0.04	0.10
n	678	676	666	664

* Significant at *p* < 0.05, ** Significant at *p* < 0.10. B = Coefficient; SE = Standard Errors. Controlling for method of survey (Public Place, Community organizations, and On-Line survey).

**Table 4:** Perceived impact of air quality on own health.

Variable/Statistic	Perceived Impact of Air Quality on Health	
	B (SE)	B (SE)
Intercept	7.52 * (0.39)	7.04 * (0.40)
Age	0.01 (0.01)	0.00 (0.01)
Female	0.53 * (0.21)	0.24 (0.19)
Latino	0.35 (0.24)	0.28 (0.22)
Other	0.11 (0.32)	0.00 (0.29)
Education (High School or below)		0.11 (0.19)
Own health issue		1.82 * (0.21)
Family members has a health issue		0.98 * (0.21)
Exercise regularly		−0.08 * (0.04)
Public Places	0.75 * (0.26)	0.56 * (0.24)
Community Groups	0.31 (0.25)	0.33 (0.23)
R^2^	0.03	0.22
n	688	674

* Significant at *p* < 0.05. B = Coefficient; SE = Standard Errors.

**Table 5:** Avoiding exercise, working outdoors, or running errands when air quality is “bad” or “unhealthy”.

Variable/Statistic	Currently Exercising (No. Days per Week)	Currently Working Out Doors (No. Days per Week)	Will Avoid Exercising	Job Requires Working Outdoors	Have to Run Errands Regardless of Air Quality
Mean	2.85 days	2.83 days	3.01	27%	68%
	B (SE)	B (SE)	B (SE)	B (SE)	B (SE)
Intercept	3.06 * (0.33)	2.41 * (0.33)	2.86 * (0.16)	0.54 (0.34)	−0.54 (0.31)
Age	−0.01 ** (0.01)	0.00 (0.00)	0.00 (0.00)	0.01 (0.01)	0.00 (0.01)
Female	0.42 * (0.18)	−0.89 (0.18)	0.26 * (0.09)	1.06 * (0.19)	0.04 (0.18)
Latino	−0.14 (0.21)	0.68 * (0.21)	0.21 * (0.10)	−0.54 * (0.22)	−0.44 * (0.20)
Other	0.35 (0.27)	0.76 * (0.27)	0.25 ** (0.13)	−0.32 (0.29)	−0.14 (0.26)
Public Places	0.61 * (0.22)	1.10 * (0.21)	−0.28 * (0.10)	−0.47 * (0.22)	−0.38 ** (0.21)
Community Groups	0.88 * (0.22)	0.67 * (0.22)	−0.023 * (0.11)	−0.06 (0.24)	−0.39 ** (0.20)
R^2^/Test of model fitness	0.04	0.11	0.03	62.98 *	15.35 *
n	681	683	681	687	685

* Significant at *p* < 0.05, ** Significant at *p* < 0.10. B = Coefficient; SE = Standard Errors; 1 = Never avoid exercising, 3 = sometimes, 5 = always avoid exercising.

**Table 6:** Sources of information about air pollution.

Variable/Statistic	Look Outside or at the Sky	Check Reports on TV	Can See Mountains Clearly	Smell of the Air	Internet or Online	Check Reports on Radio	Smart Phone	Check Air Quality Flags	Check Air Quality Index
Percentage reporting	80.0%	79.0%	73.3%	62.0%	56.1%	44.5%	42.4%	36.8%	35.4%
	B (SE)	B (SE)	B (SE)	B (SE)	B (SE)	B (SE)	B (SE)	B (SE)	B (SE)
Intercept	−1.48 * (0.36)	0.09 (0.35)	−0.82 * (0.34)	−0.99 * (0.31)	−0.77 * (0.29)	1.03 * (0.32)	−0.63 * (0.31)	0.34 (0.32)	1.38 * (0.32)
Age	0.01 (0.01)	−0.01 * (0.01)	−0.01 * (0.01)	0.01 * (0.00)	0.02 * (0.00)	0.00 (0.00)	0.03 * (0.01)	0.02 * (0.01)	−0.02 * (0.01)
Female	−0.17 (0.19)	−0.27 (0.20)	0.48 * (0.19)	0.26 (0.17)	0.01 (0.16)	0.21 (0.17)	−0.36 (0.17)	−0.29 ** (0.17)	0.47 * (0.17)
Latino	−0.08 (0.23)	−1.25 * (0.23)	0.12 (0.21)	−0.08 (0.19)	−0.24 (0.18)	−1.30 * (0.20)	−0.59 * (0.20)	−0.49 * (0.20)	−0.39 ** (0.20)
Other	−0.17 (0.32)	−0.83 * (0.30)	−0.19 (0.28)	−0.44 ** (0.26)	−0.44 ** (0.25)	−1.44 * (0.20)	−0.53 * (0.25)	−0.37 (0.25)	−1.10 * (0.26)
Public Places	−0.09 (0.24)	0.29 (0.24)	−0.09 (0.23)	−0.24 (0.21)	−0.18 (0.20)	−0.07 (0.21)	−0.31 (0.21)	−0.27 (0.21)	−0.19 (0.21)
Community Groups	−0.42 ** (0.24)	−0.07 (0.25)	0.08 (0.22)	−0.03 (0.20)	−0.028 (0.19)	0.12 (0.20)	−0.41 ** (0.21)	−0.32 ** (0.21)	−0.19 (0.21)
Test of model fitness	6.75	36.85 *	17.56 *	21.51 *	33.72 *	64.89 *	103.97 *	45.90 *	35.79 *
n	682	683	681	687	685	679	677	677	682

* Significant at *p* < 0.05, ** Significant at *p* < 0.10. B = Coefficient; SE = Standard Errors.
